# Thermal Analysis of a Disposable, Instrument-Free DNA Amplification Lab-on-a-Chip Platform

**DOI:** 10.3390/s18061812

**Published:** 2018-06-04

**Authors:** Tamás Pardy, Toomas Rang, Indrek Tulp

**Affiliations:** 1 Thomas Johann Seebeck Department of Electronics, Tallinn University of Technology, 12616 Tallinn, Estonia; toomas.rang@ttu.ee; 2 Selfdiagnostics Deutschland GmbH, 04103 Leipzig, Germany; indrek.tulp@selfdiagnostics.com; 3 Institute of Technology, University of Tartu, 50411 Tartu, Estonia

**Keywords:** Lab-on-a-Chip (LoC), finite element modelling, resistive heating, Point-of-Care (PoC), temperature control, computer aided design, microfluidics, isothermal nucleic acid amplification tests, NINAAT, NAAT

## Abstract

Novel second-generation rapid diagnostics based on nucleic acid amplification tests (NAAT) offer performance metrics on par with clinical laboratories in detecting infectious diseases at the point of care. The diagnostic assay is typically performed within a Lab-on-a-Chip (LoC) component with integrated temperature regulation. However, constraints on device dimensions, cost and power supply inherent with the device format apply to temperature regulation as well. Thermal analysis on simplified thermal models for the device can help overcome these barriers by speeding up thermal optimization. In this work, we perform experimental thermal analysis on the simplified thermal model for our instrument-free, single-use LoC NAAT platform. The system is evaluated further by finite element modelling. Steady-state as well as transient thermal analysis are performed to evaluate the performance of a self-regulating polymer resin heating element in the proposed device geometry. Reaction volumes in the target temperature range of the amplification reaction are estimated in the simulated model to assess compliance with assay requirements. Using the proposed methodology, we demonstrated our NAAT device concept capable of performing loop-mediated isothermal amplification in the 20–25 °C ambient temperature range with 32 min total assay time.

## 1. Introduction

Despite a significant drop in mortality rates associated with infectious diseases since 1990, they still constituted over 80% of all health hazards in the last decade worldwide [1]. Pathogens such as sexually transmitted diseases often have a late onset of symptoms and can have severe complications, such as infertility [2]. To prevent or effectively contain outbreaks, it is essential to have Point-of-Care (PoC) diagnostics that comply with the ASSURED (affordable, sensitive, specific, user-friendly, rapid, equipment-free and delivered) guidelines of the World Health Organization (WHO) [3]. Novel PoC tests are compliant with these guidelines and are capable of executing nucleic acid amplification tests (NAAT) in a Lab-on-a-Chip (LoC) format. These devices offer comparable performance to tests at clinical laboratories but in a far smaller and more portable package [4,5,6]. LoC NAAT tests can help decentralize testing for infectious diseases, enabling a prompt diagnosis at small clinics or even at home, as well as faster treatment of infections and better containment of pathogens. All NAAT protocols require temperature regulation to sustain the amplification reaction, but unlike the gold-standard PCR (polymerase chain reaction) protocol, isothermal NAAT protocols do not require thermal cycling, only a single reaction temperature (typically in the 20–70 °C range) maintained for the assay duration [7,8,9], which makes them favorable for portable implementations. However, their realization in a compact PoC device, especially a disposable one, is still very challenging due to space, power and cost constraints on the heating element and power supply [5]. These factors limit commercialization efforts and thereby availability to the public.

A compact, self-contained, instrument-free LoC NAAT device that meets the ASSURED criteria must have no external component and must be as small and compact as possible. However, existing demonstrations in the literature were primarily instrumented devices and relied on external means for temperature control, most commonly thermostat-regulated Peltier elements, such as those in PCR thermal cyclers [10,11,12,13]. The use of third-party external thermoregulation places end-user device price in the 1000–10,000 EUR range. The Alere I-Influenza A&B [14] system is a prime example of currently available commercial LoC NAAT devices: It consists of a stationary unit, which includes the heating system (powered from the supply grid) as well as disposable single-use microfluidic cartridges that handle the sample and execute the NAAT protocol. These benchtop instruments are not portable or instrument free, as well as costing about 10,000 EUR. Certain integrated temperature control solutions in the literature employed electrical heating elements attached to the LoC component or built into the chip itself, using thin film heaters or micro-Peltier cells [15,16,17,18,19,20,21]. However, both of these options required an external thermostat, resulting in a minimally-instrumented device rather than a completely equipment free one. Having to use microfabrication along with the required supporting electronics, the end-user price for these systems could range from 500–5000 EUR, plus 100–200 EUR for each replaceable fluidic cartridge. Self-regulating electrical heaters offer an attractive alternative in that they require no additional components for regulation [22,23,24] and rely on the PTCR (positive temperature coefficient of resistance) effect for control. Self-regulating heaters will limit their input current above a specific set temperature defined by material properties, in turn limiting their heat output. However, due to the open-loop nature of this control method, the thermal system must be carefully adjusted to expected operating parameters, as well as have a generally lower power efficiency than thermostat-regulated heaters [24]. Cost is an important design factor in instrument-free LoC NAAT devices. Exothermic chemical reactions [25,26,27] offer a very cost-effective approach to integrated heating. Oxidative reactions, such as the corrosion of magnesium-iron alloy release a significant amount of heat, in which heat release can be controlled by the addition of phase-change materials that melt in the target temperature range and dissipate excess heat. A single reaction could be done from 1–10 EUR. However, while cost effective and instrument free, the heat output of these reactions is also hard to regulate and chemical heaters tend to have a larger footprint than electrical counterparts with a similar heat output. The design complexity and higher development risk associated with chemical heaters make them less favorable for commercial devices. Common electrical heating options reported in the literature are categorized in Table 1, comparing chip dimensions, power consumption and estimated end-user price as well as whether a thermostat is required for thermal regulation. Furthermore, we assess whether the demonstrated solutions are self-contained, that is, every system component is housed within a single enclosure and the device is completely free of external instrumentation (including an external power supply). Comparing these parameters, it is apparent that self-regulating PTCR heating elements offer an ideal mix of dimensions, power consumption, production cost as well as portability for disposable LoC NAAT applications. Moreover, they enable complete device integration into a single package that is disposable as a unit. 

In previous works, we demonstrated the methodology for simulated and experimental thermal analysis of LoC NAAT systems with integrated temperature control based on commercially available electrical heating elements [30,31,32,33]. We compared the performance of a commercially available self-regulating heating element to a thermostat-regulated etched foil heating element in LoC NAAT prototypes and concluded that self-regulating heating was the favorable option for disposable instrument-free LoC NAAT devices [33]. Furthermore, we demonstrated a self-regulating heater integrated with a LoC prototype system, with which we performed loop-mediated isothermal amplification (LAMP) in the 20–25 °C room temperature range [34]. In this work, we introduce the concept device of our instrument-free LoC NAAT platform and perform thermal analysis on it to verify that it is capable of supporting LAMP in the expected end-user environment. Experimental thermal characterization is performed on a 3D-printed thermal model. The model is then simulated via finite element modelling and both steady-state and transient thermal analysis are performed. Steady-state thermal analysis is used to determine reaction temperatures at specific ambient temperatures. Transient thermal analysis is performed at the most appropriate ambient temperature to simulate reaction temperatures over time. In both cases, reaction volumes in range will be estimated based on temperature distributions and used to assess expected LAMP performance and compliance with assay requirements. 

## 2. Materials and Methods

### 2.1. Heating Element and Thermal Interface

Self-regulating resistive heating elements are electrical heaters capable of maintaining their set temperature without external regulation. PTCR (positive temperature coefficient of resistance) materials exhibit a rapid rise in resistance above a pre-determined threshold temperature and thereby limit their own input current. This allows precise temperature control within a well-defined ambient temperature range. In this paper, loop-mediated isothermal amplification (LAMP) is the protocol of choice, which provides the maximum yield within 60–65 °C temperature [7]. In our LAMP assay (detailed in our previous works [35,36,37]), we use Bsm polymerase, whose activity is higher than 80% within the 57–62 °C reaction temperature range, as demonstrated by Oscorbin et al. [38]. Therefore, we define this temperature range as the target for our heating system. Total assay time is defined as 30 min, and 10 min is allowed for the heater to bring the reaction volume up to range (the assay requires at least 20 min in the correct range to finish with certainty). BM128 batch PTCR polymer heaters (Heatron Inc., Leavenworth, KS, USA) were designed with these parameters in mind (further information on BM series heaters is available in our previous work [34]). The heaters were designed for a 3V_DC_ driving voltage supplied by 2xAAA alkaline batteries. The nominal set temperature of the heaters was 66 °C (±2 °C) to ensure fast convergence to target. 

**Figure 1:**
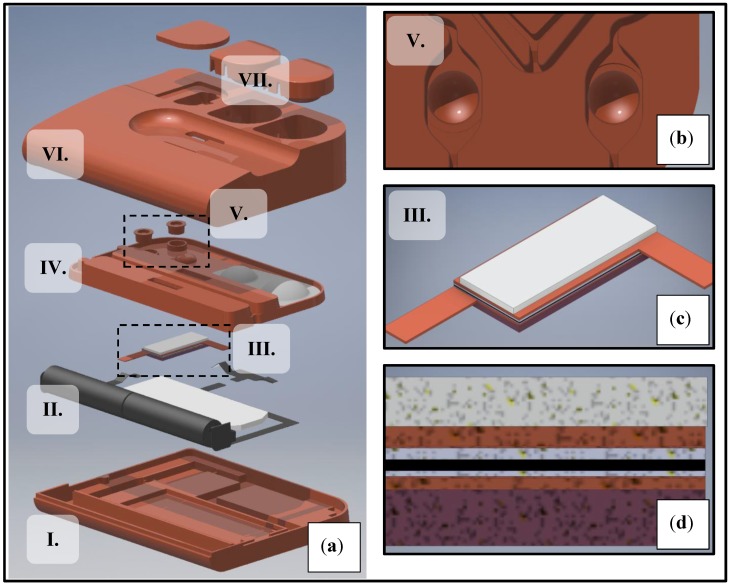
Rendering of experimental setup showing prototype components (**a**). The setup consisted of the following components: An enclosure (I., VI.) with user interface features (VII.), a microfluidic chip (IV.) with reactor chambers holding a total of 0.1 mL volume (V.) and the Heatron BM128 self-regulating heater in a sub-assembly (III.) connected to its power supply (II.). The reactor chambers (**b**), the heater (**c**) and the layer structure of the heater (**d**) are shown separately in magnified view.

The heater was built into a sub-assembly, which consisted of several layers (Figure 1c,d): The base layer was a 1.3 mm thick block of polyurethane foam (P/N 798-9310, RS Components, Corby, UK) with an adhesive layer facing the lower contact. The heater was attached with conductive PSA (pressure-sensitive adhesive) between the lower and upper contact. Both contacts were milled from 0.35 mm thick high-conductivity copper (P/N 680-959, RS Components, Corby, UK) sheets. Between the top contact and the reaction chambers was a block of acrylic foam (P/N 686-1107, RS Components, Corby, UK), self-adhesive on both sides, which conducted just the right amount of heat to the reaction liquid. The thermal interface between the heater and the heated liquid consisted of 4 layers with various thermal properties (summarized in Table 2). Therefore, parameters of this layer structure had to be optimized carefully to match operating conditions. The reaction liquid and the heater sub-assembly were separated by a 0.15 mm thick foil layer laminated onto the chip (Greiner MTP Sealers, Greiner Holding AG, Kremsmunster, Austria). No additional thermal interface material was used as the assembly was adhered to the microfluidic chip.

### 2.2. Thermal Modelling

The thermal model detailed in this work is usable for both steady-state and transient thermal analyses, given an experimentally characterized resistive heating element and a 3D device geometry with well-defined structural materials and boundary conditions. The core of the model is the heater, which generates a volumetric heat flux Q via Joule heating:

(1) $$Q=J\cdot E\;\nabla J=Q_{j}\;J=\left(\sigma +\epsilon_{0}\epsilon_{r}\frac{\partial }{\partial t}\right)E\;E=-\nabla V$$

where J, E, Q_j_ and V are the current density, electric field, current sources (sinks) and potential drop in the heating element, respectively. σ characterizes the temperature-dependent conductivity (reciprocal of resistivity) of the PTCR polymer resin in the heating element (calculated from the resistance model in Section 3.1). The generated heat is propagated in the device via the heat transfer equation, assuming zero flow (during amplification the reaction liquid is stationary). Structural materials in the simulated geometry are characterized electrically (conductivity, relative permittivity) and thermally (density, heat conductivity, specific heat capacity), as detailed in Table 2. Boundary conditions and initial values applied to the model are listed in Table 3. The experimental device was tested in a climate chamber, which provided a constant ambient temperature. Therefore, ambient temperature in the model was also considered constant. 

The model was implemented in COMSOL^®^ Multiphysics version 5.3 using the Heat Transfer and Electric Currents interfaces coupled through the Joule Heating interface. The model was solved via the built-in stationary and time-dependent solvers of COMSOL on a PC with a Core i7-7700 CPU with 32 GB RAM. Three-dimensional model geometry (Figure 1) was imported from Autodesk Inventor. Convective, conductive and radiative heat losses were taken into account in the model. All physical domains of the experimental device were taken into account in the simulation, but the geometry of the device was defeatured to decrease mesh complexity. The temperature sensor in the physical prototype was modelled by a Domain Point Probe. COMSOL generated a tetrahedral mesh of 3,663,651 elements with an average element quality (based on the well-known radius ratio method [39]) of 0.65 and an element size of 0.05 mm^3^. 

### 2.3. Experimental Setup for Thermal Characterization 

The experimental setup was a thermal mock-up (8 cm × 10 cm × 2 cm) for the instrument-free isothermal NAAT LoC platform currently under development in our labs (Figure 1a). The prototype included a microfluidic chip (5.5 cm × 9 cm × 0.75 cm) with 2 microreactors with 0.05 mL volume each (Figure 1b), and the adjacent microchannels. The fluidic chip had 4 fluidic connections (2 inlets, 2 outlets) and was filled with distilled water for the experiments through its inlets, after which the fluidic I/O features were taped over. The reaction chambers were sealed by plastic caps attached with thermoplastic adhesive. One cap had the K-type thermocouple attached to it via a hole drilled through the plastic cap and sealed with adhesive. The chip was interfaced with the heater sub-assembly mentioned in Section 2.1 (Figure 1c). The device prototype included 2xAAA alkaline batteries connected with the heater via steel clips. In the end-user device a switch would be part of the circuit, but in the prototype the switch gap was bridged by a wire. A 3 V LED backlight module (23 mm × 60 mm, from Dayear Electronic Co., Ltd., Hong Kong, China) was also part of the circuit, connected in parallel with the heater. In the end-user device, this LED would illuminate the lateral flow strips situated above it in the microfluidic chip, in order to increase contrast during readout. The device enclosure consisted of a bottom and a top part, held together by a snap-fit design. In the final device, the top enclosure would include user I/O interfaces (e.g., buttons to actuate liquids) to the chip. In the prototype, interfacial features were simplified models. 

Plastic device components were 3D printed by an SLA 3D printing system (Envisiontec Perfactory XL, Envisiontec GmbH, Gladbeck, Germany), and the channels of the microfluidic chip were sealed with Greiner MTP sealers (Greiner Holding AG, Kremsmunster, Austria). Metal clips were milled by a DATRON M7 (Datron AG, Mühltal, Germany) NC milling machine and then formed manually where needed. 

## 3. Results and Discussion

### 3.1. Characterization of Heating Element

**Figure 2:**
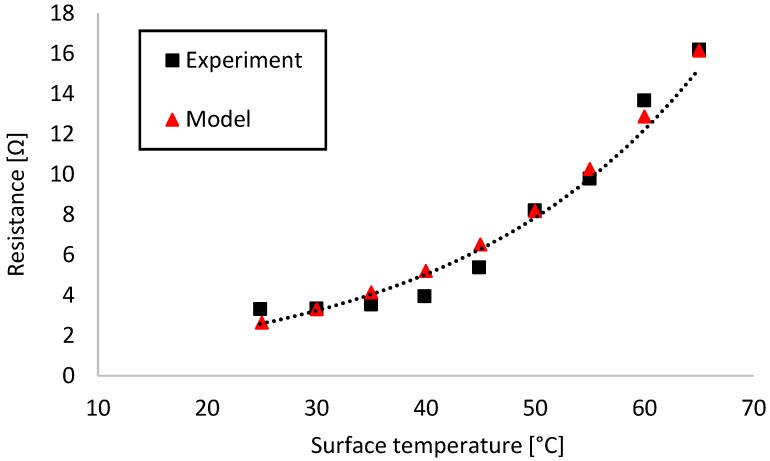
Temperature-dependent resistance profile of BM128 PTC polymer heating elements. The self-regulating heater was characterized by measuring its surface temperature and current input while receiving a steady 3V_DC_ input. The model was found by exponential curve fitting.

To get an accurate model for our thermal system, the temperature-dependent resistance profile of the self-regulating heating element had to be recorded. To this end, we set up the heater with electrical contacts in between sheets of low-density polyurethane foam (P/N 798-9310, RS Components, Corby, UK) and attached a K-type thermocouple sensor on top. The heater was powered from an Agilent E3631A triple-channel DC power supply (Agilent Technologies Inc., Santa Clara, CA, USA). Temperature was recorded with a TENMA 72–7715 digital multimeter (Tenma Test Equipment, Springboro, OH, USA). Resistance was calculated from the input current values recorded by the power supply at a stable 3 V input with 2 A current limit. Numerical data in this work was recorded with double-digit precision, except for temperature data where the measurement instrument was limited to single-digit precision. Figure 2 shows the relation between heater surface temperature and resistance. 

Exponential regression analysis was performed on the data in Microsoft Excel and yielded the following expression:

(2) $$R=0.85\cdot e^{0.0453\cdot T}$$

where T [°C] was the surface temperature of the heating element and R [Ω] was its resistance. The model showed a good fit with the experimental data (R^2^ = 0.94). Resistivity values required by the simulation were calculated from Equation (2) using the well-known formula for resistors with uniform cross-section. 

Temperature recording experiments were conducted on a 3D printed physical prototype placed in a Vötsch VT 7004 (Vötsch Industrietechnik GmbH, Bailingen, Germany) climate chamber to ensure a constant ambient temperature. Temperature inside the device prototype was recorded with a TENMA 72–7715 digital multimeter (Tenma Test Equipment, Springboro, OH, USA). The device and the multimeter were placed in the climate chamber and the ambient temperature set, after which the system was allowed 30 min to reach steady state. Then the chamber was briefly opened to insert the batteries and start the test. Reaction temperature was recorded for 30 min, after which the prototype was disassembled and allowed to reach room temperature before the next test cycle. The experiment was performed in the 15–30 °C ambient temperature range with 5 °C steps, each time with a fresh set of batteries to ensure repeatability. 

Table 4 summarizes heat-up times to the target range as well as time constants and the estimated power consumption of the heating element, whereas Table 5 summarizes steady-state reaction temperatures in the device. Power consumption P [W] was calculated from the well-known P = U^2^/R expression, where U = 3 V and R was calculated from Equation (2). The time constant for the thermal system in the device was ~27 min. Test results indicated that the reaction liquid in the device reached the correct steady-state reaction temperature range when ambient temperatures were between 20–25 °C, which is typically considered room temperature. Below this range it would take too long to reach target range, whereas above there would be a risk of overheating the reaction. Heat-up times were within the required 10 min only at 25–30 °C. The ideal ambient temperature was measured to be 25 °C, at which the steady-state temperature was 59.9 ± 0.05 °C, and the 57 °C target was reached in 8.5 min, both compliant with assay specifications. 

### 3.2. Steady-State Thermal Analysis 

**Figure 3:**
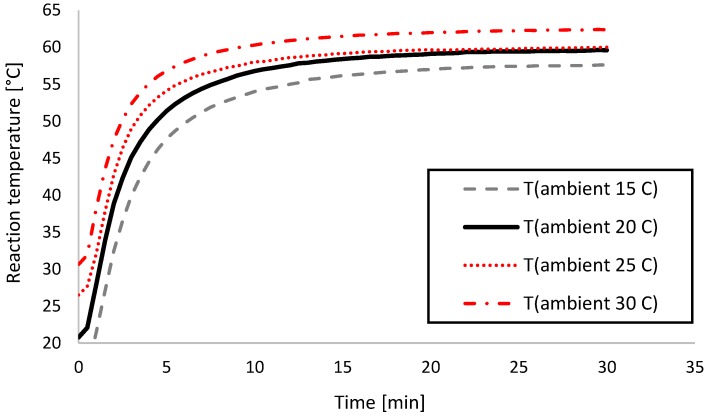
Thermal transient recordings for the experimental prototype. Each recording was conducted for 30 min in a pre-heated climate chamber.

To assess heating performance in the designated operating ambient temperature range, steady-state thermal analysis was conducted. Initially, the simulated thermal model was verified by comparing to the experimental data. Next, steady-state performance was estimated by calculating the ratio of the reaction volume in range versus the total volume. Steady-state temperature was defined as the mean of the last 5 min of the recorded data points, where variation was negligible (<0.1 °C standard deviation). Steady-state temperatures were calculated from the experimental data shown in Figure 3. 

The model was set up as described in Section 2.2 and solved using the Stationary Solver of COMSOL^®^ Multiphysics. Running a parametric sweep with all four ambient temperature points, the solution time was 48.5 min. The mean absolute error (MAE) between experimental and simulated data points was 0.4 °C. Generally, models tend to overestimate heat output in simulated thermal systems due to the fact that simulated thermal interfaces are ideal and losses are typically underestimated. In this model, this tendency was observed beyond 20 °C as the model increasingly overestimated real data. The effect was compounded by the complexity of the heater assembly (air pockets potentially stuck between self-adhesive layers). 

**Figure 4:**
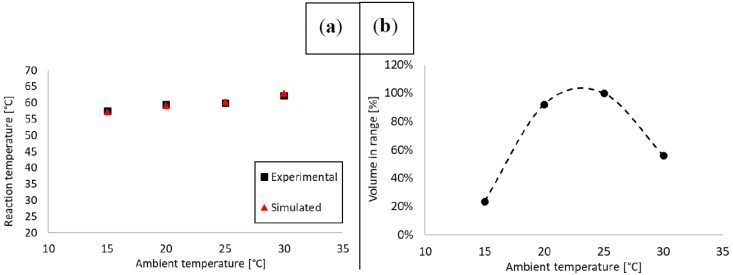
Experimental and simulated steady-state temperatures (**a**) as well as estimated reaction volume in range (**b**). Data points were recorded in the 15–30 °C temperature range with 5 °C steps, corresponding to the full ambient range expected at the end-user side (homes and offices). Based on our assessment, the device would perform best within 20–25 °C, which is commonly referred to as room temperature. At 25 °C, 100% of the reaction liquid was in range in steady-state. Below 20 °C, the test would require longer than 30 min to finish, whereas above 30 °C, polymerase activity would be insufficient, likely leading to false negatives.

LAMP is a very robust amplification protocol, it is well suited for use in an LoC setting, and provides excellent yield as long as it is run at the correct reaction temperature [7]. In our previous paper [34], we proved that when at least ~85% of the reaction liquid was in range in the chip, the reaction would complete successfully. Given that the microreactor geometry as well as the heater surface area and alignment relative to the reactors were unchanged in the chip used in this paper, we worked with the same assumption. Therefore, we estimated the percentage of the reaction liquid in range using the methodology demonstrated in our previous work [33], evaluating the logical condition (T>56.99 [°C]∩ T<62.01 [°C])∈{0;1} for each finite element within the microreactor cavity of our COMSOL model. This can be expressed with the following formula:

(3) $$\eta =100\cdot \frac{1}{n}\overset{n}{\underset{i=1}{\sum }}\left(\left(T_{i}>56.99\;\left[{}^{\circ}C\right]\cap \;T_{i}<62.01\;\left[{}^{\circ}C\right]\right)\in \left\{0;1\right\}\right)$$

where T_i_ denotes temperature values for each finite element, n the total element number and η∈[0;100]. Table 5 summarizes temperature data and estimated reaction volumes in range at the ambient temperatures specified in the previous section. Figure 4 shows a graphical comparison between experimental and simulated reaction temperature data, as well as reaction volume in range relative to ambient temperature. 

To summarize, steady-state thermal analysis was conducted on our concept device at 15–30 °C ambient temperatures with 5 °C steps. The device was modelled with a simulation to reveal the volume of the reaction liquid in range. In the 20–25 °C ambient temperature range, typically considered as room temperature, the device met the target temperature criterion in our experiments as well as had an estimated 90–100% of the reaction liquid in the range based on our models. Heating efficiency peaked at 25 °C ambient temperature. Below 20 °C the reaction would need considerably more time to finish than 30 min, and above 30 °C the activity of the Bsm polymerase would diminish significantly until it became completely inactivated. Therefore, our proposed device design was proven valid for use in the 20–25 °C ambient temperature range (common in homes and offices) with respect to steady-state temperatures. 

### 3.3. Transient Thermal Analysis

As described in the previous section, the steady-state temperature target stated in Section 2.1 was met in the 20–25 °C ambient temperature range. The other assay criterion to meet was the rise time to the target range (10 min maximum) and the total time in range (20 min minimum). All these criteria were only possible to meet at 25 °C ambient temperature, which we analyzed further with transient thermal analysis. First, we took the experimentally recorded thermal transients shown in Figure 3 and compared them to the output of the simulation to verify their accuracy. Then we calculated Equation (3) for all simulated time points to determine the percentage of the reaction volume in range. 

**Figure 5:**
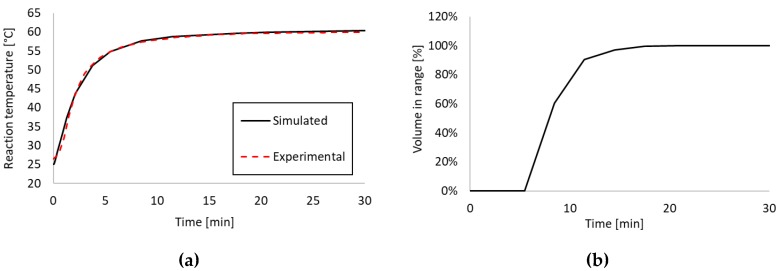
Experimental and simulated thermal transients for the reaction temperature in the device (**a**) as well as estimated reaction volume in range in time (**b**). Data points were recorded and simulated at 25 °C ambient temperature.

The model was once more set up as described in Section 2.2 and solved using the Time-Dependent Solver of COMSOL^®^ Multiphysics. Solution time was 2 h 39 min for a simulated transient of 30 min. The MAE between experimental and simulated data points was 1.2 °C at 25 °C ambient temperature, and the model estimated both the heat-up time and the time constant accurately. Figure 5 shows the results of the transient thermal analysis. 

As stated previously ~85% of the reaction volume (in the reaction chambers) had to be in the target temperature range 57–62 °C for the reaction to be successful. Analyzing the percentage of the reaction volume in range using Equation (3), we can conclude that within 10 min the required ~85% of the volume was in range. With the current device concept and assay requirements, the device would work most reliably at 25 °C ambient temperature. While the temperature sensor measured 57 °C already at 8.5 min, it took ~10 min for the majority of the reaction volume to reach the target range. Considering this 1.5 min difference, the operating ambient temperature range of the device could easily be extended to include 20 °C with one of two options: (1) Extending the total assay time to at least 32 min, (2) adding additional insulation. Option (2) is not easily practicable for a well-established system given that any added insulation takes up additional space, changes the thermal system and mandates a design overhaul. Option (1) is far easier to implement and is an acceptable trade-off to ensure that no false negatives occur on the end-user side. 

## 4. Conclusions

In this work we demonstrated a thermal analysis methodology for the evaluation of LoC NAAT devices. The proposed process was demonstrated on a simplified thermal model for an instrument-free single-use LoC NAAT platform with integrated temperature control via a self-regulating polymer resin heater. The LoC component was designed to house a LAMP reaction for chlamydia trachomatis (CT), established in our previous works. This assay required 57–62 °C reaction temperature and at least 20 min incubation time. Therefore, experimental and simulated steady-state thermal analysis was performed to establish compliance with the temperature target at ambient temperatures between 15–30 °C with 5 °C steps. Our proposed finite element model estimated steady-state temperatures with 0.4 °C error and was used to estimate reaction volumes in range in steady-state to verify assay compliance. In our previous work we demonstrated that at least ~85% of the reaction liquid had to be in range for a successful reaction. This condition was met in the 20–25 °C ambient temperature range. Thermal transients were recorded for the experimental thermal model and determined that the test would perform best at 25 °C ambient temperature. The simulated thermal transient was within 1.2 °C of the experimental record and was used again to estimate the reaction volume in range over time. It was determined that ~85% of the reaction volume was in range after 10 min and thus the 20 min incubation time criterion was possible to meet with 30 min assay time. However, to extend the operating ambient temperature range of the device to the 20–25 °C room temperature range, the total assay time would need to be extended to 32 min. 

Summarily, we demonstrated a comprehensive thermal analysis methodology to verify assay compliance in LoC NAAT devices and performed the analysis to demonstrate our proposed LoC NAAT platform capable of executing our CT-LAMP assay at 20–25 °C ambient temperature under 32 min. The demonstrated heating solution offers a unique combination of power-efficiency (~1 W max., on 2xAAA alkaline batteries), small size (1 cm × 2 cm × 0.5 cm), low cost (~20–50 EUR end-user device price) and good disposability (no toxic structural materials). Furthermore, it is realized by using only passive electronic components in a minimalistic and fully self-contained design that enables disposal as a unit after each use. This set of features address several of the barriers to commercializing LoC NAAT systems, and it is our hope that this contribution will bring affordable homecare DNA analysis along with its benefits to preventive medicine one step closer. 

## 5. Patents

Selfdiagnostics Deutschland GmbH has a pending patent application (PA201770310) that details the integration of polymer resin heating elements into Lab-on-a-Chip devices.

## Figures and Tables

**Table 1:** Comparison of electrical heating solutions for LoC NAAT devices reported in the literature.

Heating Solution	Self-Contained?	Thermostat?	Chip Dimensions [cm]	Power Consumption [W]	End-User Device Price (Estimate) [€]	Source
External Peltier	No	Yes	~4 × 4 × 2	min. 80	~1000–10,000	[10,11,12,13]
Integrated resistive	Examples available	Yes	~8 × 3 × 1	~2–3	~500–5000	[15,16,17,18,19]
Integrated μ-Peltier	No	Yes	~2 × 2 × 0.3	~2–3	~500–5000	[20,21,28]
Integrated self-regulating	Not reported	No	~5 × 5 × 0.3	~1.2–0.8	~500–1000	[24,29]
Integrated polymer resin PTCR	Yes	No	5.5 × 9 × 0.75	1–0.6	~20–50	This work

**Table 2:** Summary of material properties used in the model.

Material	Density [kg/m^3^]	Thermal Conductivity [W/mK]	Specific Heat Capacity [J/kgK]	Electrical Conductivity [S/m]
PTCR heater resin	2250	1.26	1000	0.3–0.06 (20–65 °C range)
Aluminum	2700	238	900	37.7 × 10^6^
Copper	8960	400	385	59.9 × 10^6^
Polycarbonate	1200	0.14	1250	Not relevant
Air	1225	0.024	1000	Not relevant
Water	1000	0.6	4184	Not relevant
Polyurethane	1250	0.3	1760	Not relevant
Acrylic foam	850	0.18	1470	Not relevant
Paper (LF strip)	1500	0.05	1340	Not relevant
Steel	7850	44.5	475	4.03 × 10^6^

**Table 3:** Summary of boundary conditions and initial parameter values used in the model.

Boundary Condition	Boundary	Initial Value (If Applicable)
Ambient temperature	External boundaries	As defined in text
Ambient pressure (absolute)	External boundaries	1 atm
Electric potential	Heater (top)	3 V
Ground	Heater (bottom)	0 V
Convective heat loss	External boundaries	Not applicable
Electrical insulation	Heater boundaries except contacts	Not applicable
Radiative heat loss	External boundaries	Not applicable
Joule heating (boundary)	Heater boundaries	Not applicable

**Table 4:** Summary of heat-up times and time constants for the recorded thermal transients, as well as power consumption of the heating element.

Ambient Temperature [°C]	Heat-Up Time to 57 °C [min]	Time Constant [min]	Power Consumption [W]
15	20	27.5	0.94
20	10.5	27	0.86
25	8.5	27.5	0.84
30	5.5	26	0.76

**Table 5:** Summary of experimental and simulated steady-state reaction temperatures and reaction volume in range values in the 15–30 °C ambient temperature range with 5 °C steps.

Ambient Temperature [°C]	Experimental Steady-State [°C]	Simulated Steady-State [°C]	Reaction Volume in Range [%]
15	57.5	57.3	24
20	59.5	59.2	92
25	59.9	60.3	100
30	62.3	63.1	56
